# Gratitude Moderates the Relation between Daily Hassles and Satisfaction with Life in University Students

**DOI:** 10.3390/ijerph182413005

**Published:** 2021-12-09

**Authors:** Guillaume Tachon, Rebecca Shankland, Fanny Marteau-Chasserieau, Blaire Morgan, Christophe Leys, Ilios Kotsou

**Affiliations:** 1 Laboratory DIPHE (Développement, Individu, Processus, Handicap, Education), Institut de Psychologie, Université Lumière Lyon 2, 5 Avenue Pierre Mendès-France, 69676 Bron, France; 2 Laboratory VCR (Vulnérabilité, Capabilité, Rétablissement), Ecole de Psychologues Praticiens, 23 rue Montparnasse, 75006 Paris, France; fmarteauchasserieau@psycho-prat.fr; 3 School of Psychology, University of Worcester, Worcester WR2 6AJ, UK; b.morgan@worc.ac.uk; 4 Centre de Recherche en Psychologie Sociale et Interculturelle, Université Libre de Bruxelles, Avenue Franklin Roosevelt 50, 1050 Bruxelles, Belgium; chleys@gmail.com (C.L.); ilios.kotsou@gmail.com (I.K.)

**Keywords:** gratitude, satisfaction with life, daily hassles, gratitude questionnaire, students

## Abstract

Satisfaction with life as a judgmental cognitive process can be negatively influenced by appraisals of daily events such as hassles. Trait-gratitude—a tendency to appraise, recognize and respond to life events through being grateful—is a determinant of mental health and well-being, and has been shown to be related to the positive appraisal of life. The aim of the current study was to investigate the moderating role of trait-gratitude in the relationship between daily hassles and satisfaction with life. In the process of carrying out this study, the French version of the Gratitude Questionnaire (GQ-6) was validated. A total of 328 French undergraduates completed questionnaires measuring gratitude, satisfaction with life, and daily hassles to test the main hypothesis. They also completed optimism, coping strategies, depression, and anxiety questionnaires in order to assess the convergent validity of the French version of the GQ-6. First, the results showed satisfactory psychometric properties of the Gratitude Questionnaire. Second, the results indicated the moderating role of trait-gratitude in the relationship between daily hassles disturbance and satisfaction with life. This study further documents the role of gratitude as a determinant of well-being and provides French-speaking clinicians and researchers with a useful tool to measure grateful disposition.

## 1. Introduction

University students have been shown to be more vulnerable to the onset of common mental health problems and psychological distress compared with the age-matched general population [1]. First years at university represent a period which may threaten students’ mental health and well-being [2], and requires using active coping strategies to adapt to a new context [3]. This transition leads to a host of stressors such as leaving home, reduced social support, academic pressure and decision-making challenges [4]. Research has shown that such daily hassles reduce students’ well-being [5].

Students have to cope with strains, related to new environments, roles, workloads and relationships, which can be hassling [5]. Daily hassles are defined as ‘experiences and conditions of daily living that have been appraised as salient or harmful or threatening to the endorser’s well-being’ [6] (p. 376). Hassled people make a subjective and negative judgment of daily events [6]. Even if hassles are directly related to an objective and harmful experience, the meaning inferred by people leads them to remember it in a more salient and distressful way [6]. University students tend to use emotion-focused coping strategies such as avoidance and self-punishment to deal with daily hassles, which is associated with negative affect and has a negative influence on health [7,8]. Therefore, daily hassles are positively related to physical illness [9] and to psychopathological symptomatology such as stress and burnout [10,11]. Hassles are also negatively associated with life satisfaction [12]. Thus, daily hassles negatively influence mental health and well-being.

This influence could be understood by considering how people appraise their subjective well-being. According to Diener’s conceptualization [13], subjective well-being is composed of (1) a cognitive judgment of overall life satisfaction, and (2) of emotions. Life satisfaction reflects a judgmental evaluation of one’s life [14]. This cognitive process relies on a comparison of one’s perceived conditions of living with one’s own targeted standard of life [13]. Therefore, daily hassles and life satisfaction are two appraisals of daily living conditions. In the academic context, improving subjective well-being is an issue that could be dealt with by reducing students’ evaluation of daily hassles.

One means of reducing the influence of daily hassles on life satisfaction could be through a grateful outlook towards life. According to McCullough, Emmons, and Tsang [15], trait-gratitude is defined as ‘a generalized tendency to recognize and respond with grateful emotion to the roles of other people’s benevolence in the positive experiences and outcomes that one obtains’ (p. 112). In this conceptualization, the grateful disposition is built on the cognitive processes of benefit appraisal and its characteristics appraisal (e.g., the cost to the benefactor), and on the emotional process of appreciation of the benefit [16]. Gratitude is negatively related to negative affect, depression, and anxiety [17,18], and positively associated with well-being [19,20]. Indeed, scholars have highlighted the beneficial role of gratitude interventions on mental health and well-being, while participants included in the daily hassles condition showed no such improvement [16,21]. Considering the coping hypothesis [22], grateful individuals tend to adopt more active coping strategies, such as seeking social support or positive reframing [23]. Therefore, they are more able to positively reappraise negative events, and less likely to use self-blame [22]. This possible underlying process could explain how gratitude is related to higher levels of life satisfaction: grateful individuals frequently experience grateful thoughts, allowing them to be less distressed [23]. Given this evidence [7,8], it could be hypothesized that trait-gratitude moderates the relationship between daily hassles and satisfaction with life. However, the nature of this influence and the role of trait-gratitude are to be determined.

## 2. Overview of the Study

This study was part of a larger research project on mental health in French university students [11]. The main goal of the current study was to analyze the possible impact of gratitude on the relationship between daily hassles and satisfaction with life. Given the previous argument, we hypothesized that the influence of both frequency and disturbance of daily hassles on satisfaction with life would be weaker for students who present a higher level of trait-gratitude, suggesting a moderating role of trait-gratitude. The secondary goal was to analyze the psychometric qualities of the French version of the GQ-6, and examine its correlations with mental health, well-being and optimal functioning. To date, no French validation of any gratitude measure has been published. To assess the construct validity, we hypothesized that trait-gratitude would be positively correlated with optimism, active coping (problem-focused and seeking social support), and satisfaction with life, and negatively correlated with anxiety and depression. As suggested in the literature [22], we expected that trait-gratitude and emotion-focused coping would not covary. To study the influence of trait-gratitude on daily hassles in university students has the potential to promote more adaptive coping strategies among this specific population. If a beneficial relationship between trait-gratitude, daily hassles and satisfaction with life is shown, then it could support further research to better understand the mechanisms underlying this relation, and also support gratitude-based intervention programs to promote optimal functioning and a wider thought–action repertoire or coping strategies in university students. This study could also provide a substantial benefit to the gratitude field if reliable psychometric properties of the French version of the GQ-6 are shown. Indeed, providing a French validation of a globally used gratitude measure closes a key measurement gap and should promote gratitude research in French contexts and allow researchers to assess the effectiveness of gratitude interventions in French-speaking populations. Overall, if there are meaningful results, this research could be useful in both clinical and research domains in France.

A cross-sectional design was used to assess our hypothesis. The validation of the French version of the GQ-6 is required to test the main hypothesis. Therefore, the first part of the study aimed at assessing the psychometric qualities of the French version of the GQ-6, and the second part of the study examined the hypothesized moderation.

## 3. Part 1. Validation of the French Version of the GQ-6

### 3.1. Material and Methods

#### 3.1.1. Translation and Validation Process

Following Vallerand’s transcultural adaptation process [24], a translation and back-translation were performed. To support the validation process, confirmatory factorial analysis (CFA) was first performed. We did not perform an exploratory factorial analysis given the existing validation studies in the literature that inform the factorial structure of the GQ-6 [25,26,27]. The factorial structure and the relationships item-factor of the French version of the GQ-6 appeared through the CFA. This type of analysis allowed the evaluation of the overall model through the fit index. Then, Cronbach’s alpha was measured. Second, the construct validity was assessed through correlations with mental health indicators (depression, anxiety, satisfaction with life) and determinants (optimism and coping) which have already been shown to correlate with gratitude in past studies [18,25,27].

#### 3.1.2. Participants

From the original sample of 347 students, 10 were removed for missing data because there was at least one missing response on the GQ-6. Listwise deletion was performed given that missing data were missing completely at random (MCAR) and represented less than 5% of the data set. The sample size also allowed for the choice of listwise deletion. The nine multivariate outliers identified through Mahalanobis distance were also removed from the analysis. All analyses were performed on the remaining sample of 328 university students (269 females) in psychology, sociology, education and sports of three French universities. Age ranged from 19 to 57 (*M* = 22.67, SD = 4.12). The sample included predominantly individuals who lived not alone and had no children (see Table 1 for descriptive data). Almost half of the sample (46.3%) worked alongside university courses.

#### 3.1.3. Procedure

The paper questionnaires were administrated during academic sessions in the second part of the academic year. All students who volunteered to participate provided informed consent prior to taking part in this study, in accordance with the 1964 Helsinki declaration and its later amendments. Participants were asked to put the questionnaires back in a box. Thus, direct contact between instructor and participants was avoided.

#### 3.1.4. Measures

The measures included in this study were also used in previous validation studies of the GQ-6 [25,26,27] and their relations with GQ-6 were well informed across the literature, as for depression and anxiety for example [18]. These elements make these instruments useful and relevant to assess the construct validity.

Dispositional gratitude. Trait-gratitude was assessed using the French version of the GQ-6 [15]. Participants rated the six items (two reverse coded) on a 7-point Likert Scale ranging from 1 (strongly disagree) to 7 (strongly agree). As explained in the results section (see Section 3.2), GQ-5 showed a satisfactory internal consistency (α = 0.74). Mean score was 4.97 (SD = 0.91).

Optimism. The Life Orientation Test Revised (LOT-R) [28] was used in its French version [29] to assess trait-optimism. Participants rated a 10-item scale, on a 5-point Likert scale ranging from 0 (strongly disagree) to 4 (strongly agree). Four filler items were removed before performing analyses. The LOT-R showed a satisfactory internal consistency (α = 0.80). Mean score was 2.01 (SD = 0.73).

Coping. The Ways of Coping Checklist Revised [30] was used in its French version [31]. Participants rated the 27 items on a 4-point Likert scale, from 1 (No) to 4 (Yes), assessing problem-focused coping (M = 2.97; SD = 0.38), emotion-focused coping (M = 2.66; SD = 0.53), and seeking social support (M = 2.72; SD = 0.55). All internal consistencies were satisfactory (respectively α = 0.72, α = 0.72, α = 0.75).

Satisfaction with life. The French version [32] of the Satisfaction With Life Scale (SWLS) [14] assessed current life satisfaction through five items rated on a 7-point Likert scale (1 = strongly disagree; 7 = strongly agree). SWLS showed satisfactory internal consistency (α = 0.82). Mean score was 4.64 (SD = 1.20).

Anxiety Trait-anxiety was assessed using the French-Canadian version [33] of the State–Trait Anxiety Inventory (STAI) [34]. Twenty items were rated on a 4-point Likert scale, ranging from 1 (almost never) to 4 (almost always). The internal consistency was satisfactory (α = 0.90). Mean score was 2.35 (SD = 0.49).

Depression. The French version [35] of the Center for Epidemiological Studies Depression Scale (CES-D) [36] was used to assess depression symptoms through four subscales (i.e., negative affect, positive affect, somatic complaints, interpersonal interactions problems). Twenty items were rated on a 4-point Likert Scale, from 0 (rarely or none of the time) to 3 (most or all of the time). Internal consistency (α = 0.90) is satisfying. Mean score was 0.88 (SD = 0.49).

### 3.2. Results Part 1: French Version of the GQ-6 Structure Validity

**Figure 1:**
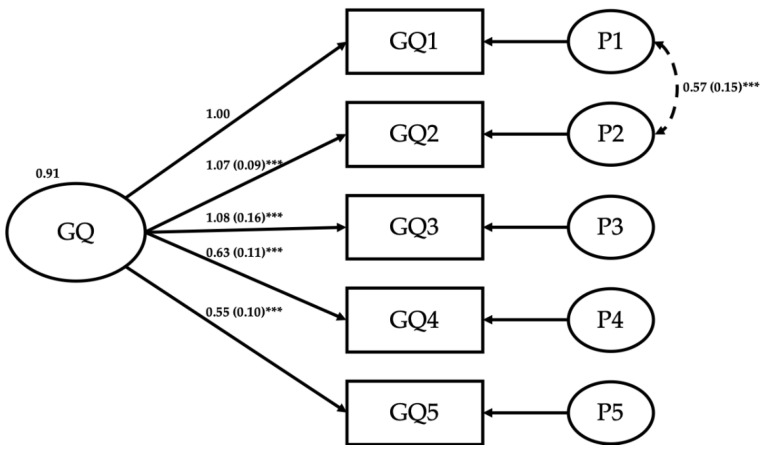
Path model of GQ-6 on full sample (N = 328). Note: GQ6 has been removed from scale due to its poor contribution to the model. *** indicates *p* < 0.001.

*French version of GQ-6 reliability*. Since we wanted first to explore the modification indices, we conducted a CFA, using Maximum Likelihood method, on two subsamples in order to adjust the model on the first subsample and to test the model invariance on the second. First, we used an r code randomly separating (with a probability of 0.5) each observation in one of two the sub-samples. We used the smallest sub-sample (N = 158) to conduct prior CFA analysis. The first model tested the validity of a model using all 6 items. It yielded an acceptable model fit (*χ*^2^/df = 1.96, df = 8, *p* = 0.04; CFI = 0.95; TLI = 0.92; RMSEA = 0.08 [0.02–0.13]). However, results showed that one of the items (OR6) did not load on the factor (b = −0.057, SE = 0.12, *p* = 0.64). Moreover, the analysis of modification indices showed that the correlation between the first and the second error terms (perturbation) of the item 1 and 2 had to be estimated (see Figure 1, double arrow between P1 and P2). We removed item OR6, set the correlation between the perturbations and conducted the analysis a second time. It yielded a very good model fit (*χ*^2^/df = 1.09, df = 4, *p* = 0.36; CFI = 0.998; TLI = 0.995; RMSEA = 0.02 [0.00–0.13]). We then conducted this last analysis on the second sub-sample (N = 170) to ensure our model (*χ*^2^/df = 1.92, df = 4, *p* = 0.10; CFI = 0.99; TLI = 0.96; RMSEA = 0.07 [0.00–0.15]). Lastly, we conducted this analysis on the full sample which yielded an acceptable model fit (*χ*^2^/df = 3.28, df = 4, *p* = 0.01; CFI = 0.98; TLI = 0.95; RMSEA = 0.08 [0.04–0.14]) although the *χ*^2^/df is slightly too high showing a limited consistency between the theoretical model and the data. However, this slight discrepancy might be attributed to a strong correlation between the first and the fourth items perturbations that was not revealed in the first subsample (although it did in the second). Figure 1 shows the path model.

*Construct validity.* The correlations between the French version of the GQ-5 and anxiety, depression, coping, life satisfaction and optimism were computed. None of the demographic or situational variables had a significant influence on the measure of dispositional gratitude.

*Convergent validity.* On the basis of previous research on gratitude, positive correlations were expected between gratitude and satisfaction with life, optimism, problem-focused and social support seeking coping, while negative correlations were expected with depression and anxiety measures. All the correlations were in the expected direction (see Table 2). Satisfaction with life, social support seeking coping and optimism showed the strongest correlation with trait-gratitude. Active coping was thus positively correlated with trait-gratitude, as expected, while there was no correlation with emotion-focused coping. Trait-gratitude was also negatively and weakly associated with symptom measures of depression and anxiety.

### 3.3. Discussion Part 1

The first step to assess the main hypothesis of the current study was to test the validity of the French version of the GQ-6. Therefore, we examined the factorial structure, the internal consistency and convergent validity, showing satisfactory psychometric properties of the French version of the GQ-5. Whereas item 6 was included in several versions of the measure [25,26], the poor contribution of item 6 has generally been mentioned [27], impeding replication and validation of the initial model of the GQ-6 in other cultures. The presence of this problem identified in various cultures supports the removal of item 6 in the current study. Furthermore, the correlation between error terms of the items OR1 and OR2 had to be estimated in the model. This covariation could be due to the fact that these items both measured the “span” facet of the grateful disposition. The French version of the GQ-5 showed a similar reliability to those reported in the literature [25]. Correlations appeared to be lower than those mentioned in past literature [15,27], but are still in line with research in this field. These results support the perspective according to which gratitude is a relevant determinant of mental health and well-being. In sum, preliminary evidence showed that the French version of the GQ-5 can be a reliable measure of gratitude in French contexts.

## 4. Part 2. The Moderating Role of Gratitude

Our main hypothesis was that trait-gratitude would moderate the relation between daily hassles and satisfaction with life. Indeed, we expected that daily hassles frequency and disturbance would have a weaker impact on satisfaction with life for those who scored higher on gratitude measure.

### 4.1. Material and Methods

#### 4.1.1. Participants and Procedure

To test our hypothesis, the same participants and procedure were used.

#### 4.1.2. Measures

The same measures of gratitude and satisfaction with life as in part 1 were used. Daily hassles frequency and disturbance measures were added.

Daily hassles. The Reveillère et al. [5] French version of the Daily Hassles Scale Revised (DHS-R) [37] was used. The DHS-R consists of 65 items rated on a 4-point Likert scale. Frequency (1: never; 4: frequent) and disturbance (1: not at all disturbed; 4: very much disturbed) of daily hassles were measured (e.g., ‘not enough money for basic necessities’, ‘too many things to do’). Mean scores were 1.18 (SD = 0.37) for the frequency scale and 1.32 (SD = 0.47) for the disturbance scale. For both subscales, internal consistencies were very satisfactory (respectively, α = 0.91 and α = 0.94).

### 4.2. Results Part 2

**Figure 2:**
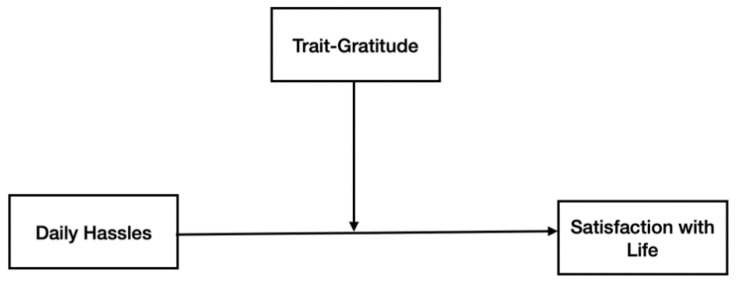
Moderation model of gratitude moderating the relation between daily hassles disturbance and satisfaction with life.

*Moderation analysis.* Prior correlation analysis showed that none of the daily hassles frequency or disturbance was significantly correlated to trait-gratitude (respectively r = −0.08, *ns*, r = 0.10, *ns*). Then, a moderation analysis could be performed. Analyses revealed that disturbance of daily hassles negatively predicted satisfaction with life; that trait-gratitude positively predicted satisfaction with life; that disturbance of daily hassles interacted with trait-gratitude (see Table 3). Therefore, trait-gratitude appears to be a strong moderator of the relation between disturbance of daily hassles and satisfaction with life (see Figure 2). No significative moderating effect of trait-gratitude was found in the relation between frequency of daily hassles and satisfaction with life.

### 4.3. Discussion Part 2

Disturbance of daily hassles was a negative and moderate predictor of satisfaction with life. Nevertheless, this relation was weaker for grateful people (i.e., people who scored high on the GQ-5). This suggests that the grateful disposition moderates and weakens the predicting effect of daily hassles disturbance on satisfaction with life. Thus, trait-gratitude appears to act as a buffer against the influence of the disturbance of daily hassles. Previous studies underlined that gratitude represents a positive resource to face adverse situations, based on underlying coping processes such as positive reframing and reinterpretation [22,23]. Our results (i.e., positive correlation between trait-gratitude and active coping strategies, see Table 2) are in line with findings suggesting that the more grateful individuals are, the more they tend to reinterpret negative events in a positive way and are able to develop personal growth through such adverse situations [22]. This way of reframing previously negatively perceived events could explain the moderating role of gratitude between disturbance of daily hassles and life satisfaction [23]. Thus, although daily hassles may be perceived as frequently by grateful individuals, trait-gratitude seems to reduce how daily hassles affect individuals’ evaluation of one’s own life.

## 5. Discussion

As a cognitive–judgmental process, satisfaction with life can be influenced by the way events are appraised [14]. In doing so, daily hassles are a threat to the well-being of individuals who appraise events in a negative manner [6]. Gratitude might function as a means of managing the effects of daily hassles. This study showed that trait-gratitude had a moderating role in the relation between daily hassles disturbance and satisfaction with life. This finding supports the literature in the field suggesting a role of gratitude disposition as a determinant of well-being [19], and provides insights as to how trait-gratitude and daily hassles influence life satisfaction. One possible explanation of these results relies in the strategies used by grateful people to cope with hassles. This perspective supports the relevance of developing gratitude to promote well-being, and investigating the processes, such as positive reframing, involved in the relation between daily hassles, trait-gratitude and life satisfaction among university students. These findings on the buffering role of trait-gratitude between daily hassles disturbance and satisfaction with life, could promote the development of gratitude-based interventions among universities. Promoting mental health is a current issue that needs to be managed, especially during this critical period of life for students [1,8]. Gratitude-based intervention could be useful to students as a way to promote more adaptive coping strategies [22,23], in addition to psychological, subjective and social well-being [19, for a review]. This type of intervention also has the advantage of being a low-cost intervention, accessible, and easy to engage, in the context of a diverse student population with restricted budgets [38]. Even if weak to moderate effect sizes of the effectiveness of gratitude interventions to reduce anxiety and depression are observed [17,20], gratitude interventions could be useful to cope with the daily annoyances and then promote students’ well-being.

The secondary aim of the study was to document the psychometric qualities of the GQ-6 and its correlations with mental health and well-being determinants and indicators in a French context. The results showed preliminary evidence of the reliability of the French GQ-5. Further investigation of the psychometric properties of this measure could be useful to make sure that it is a reliable measure of gratitude disposition. Furthermore, it is important to inform users of French GQ-5 of the fact this measure reflects one specific conceptualization of gratitude construct, which could be narrower than how the gratitude construct is understood and used by laypeople [39]. Moreover, GQ-6 assessed only feelings of gratitude. This has some limits. First, gratitude experience appears to be more complex than just feelings of gratitude, considering the behavioral component of gratitude [40] or the willingness to reciprocate as the crucial point of gratitude being understood as a moral virtue [41]. Second, discrepancies between the theoretical and operational definitions of gratitude can be noticed. While the theoretical definition focused on a triadic conceptualization (i.e., including a benefit, a benefactor, and a beneficiary), half of the GQ-6 items assessed dyadic gratitude (i.e., including a beneficiary and a benefit) [41,42]. So, if the GQ-6 has become the most widely used instrument of measure to study gratitude, we have to be aware of its limits. Based on these elements and on the fact that gratitude interventions rely on cognitive, emotional, and behavioral processes, we suggest defining trait-gratitude as a tendency to appraise, recognize and respond to life events through a grateful attitude. The term ‘attitude’ is used as a merging of emotional, cognitive and behavioral components. According to this consideration, grateful individuals tend to feel, think and behave in a more grateful way than less grateful individuals [40]. Future extensions of this work include the translation of multifaceted gratitude measures, such as the Multi-Component Gratitude Measure [40], to further the examination of gratitude in French contexts.

## 6. Limitations

The main limitation lies in the cross-sectional nature of the design, which can be useful to investigate the relation between variables. However, it prevents from concluding on causal relation between any variables measured. The self-reported nature of data also adds to this limitation. Further research built on a longitudinal or prospective design are necessary to identify causal links. This cross-sectional design also impedes from assessing the test–retest reliability of French version of the GQ-5. Further research is needed to investigate this dimension of psychometric properties of the measure in the French population. Furthermore, as this measure was carried out among a convenience sample mostly composed of female respondents, further research is required to investigate the psychometric properties of the GQ-5 in the French general population.

## 7. Conclusions

The current research contributes to the gratitude literature in several ways. First, the French version of GQ-5 showed satisfactory preliminary psychometric qualities, which makes the assessment of gratitude disposition in clinical and research domains in French contexts possible. Second, trait-gratitude among undergraduates operates as a buffer against the disturbance of daily hassles on satisfaction with life. It supports the perspective according to which gratitude represents a determinant of mental health and well-being. These promising results encourage further investigation of underlying processes at work in gratitude disposition or interventions.

## Figures and Tables

**Table 1:** Sample description.

Variable	Categories	Number of Participants	% of the Sample
Gender	Male	59	18
	Female	269	82
Level of education	Bachelor	202	61.6
	Masters	126	38.4
Living alone	Yes	130	39.6
	No	198	60.4
Having children	Yes	10	3
	No	318	97
Work alongside university	Yes	152	46.3
	No	176	53.7

**Table 2:** Pearson’s correlations between GQ-5 and indicators and determinants of mental health and well-being.

Variable	1	2	3	4	5	6	7	8
1. GQ-5 (centered)	-							
2. Satisfaction with life	0.39 **	-						
3. Optimism	0.30 **	0.51 **	-					
4. Depression	−0.24 **	−0.54 **	−0.47 **	-				
5. Anxiety	−0.25 **	−0.59 **	−0.61 **	0.70 **	-			
6. Problem-focused coping	0.20 **	0.26 **	0.37 **	−0.32 **	−0.29 **	-		
7. Emotion-focused coping	0.01	−0.37 **	−0.39 **	0.46 **	0.55 **	−0.18 **	-	
8. Social support-seeking	0.38 **	0.15 **	0.12 *	−0.18 **	−0.08	0.33 **	0.00	-

** indicates *p* < 0.001; * indicates *p* < 0.05.

**Table 3:** Linear model of predictors of satisfaction with life.

	b	SE B	t	*p*
Constant	4.64	0.056	83.44	*p* < 0.001
Gratitude (centered)	0.091	0.011	8.257	*p* < 0.001
Disturbance of daily hassles (centered)	−0.917	0.119	−7.69	*p* < 0.001
Interaction	0.061	0.02	3.017	*p* = 0.003

Note: *R*^2^ = 0.31.

## Data Availability

Data is available upon request to the corresponding author.

## References

[1] Larcombe W., Finch S., Sore R., Murray C.M., Kentish S., Mulder R.A., Lee-Stecum P., Baik C., Tokatlidis O., Williams D.A. (2015). Prevalence and socio-demographic correlates of psychological distress among students at an Australian university. Stud. High. Educ..

[2] Dyrbye L.N., Thomas M.R., Shanafelt T.D. (2006). Systematic review of depression, anxiety, and other indicators of psychological distress among U.S. and Canadian medical students. Acad. Med..

[3] Shankland R., Genolini C., França L.R., Guelfi J.-D., Ionescu S. (2010). Student adjustment to higher education: The role of alternative educational pathways in coping with the demands of student life. High. Educ..

[4] Baghurst T., Kelley B.C. (2014). An Examination of Stress in College Students Over the Course of a Semester. Health Promot. Pract..

[5] Réveillère C., Nandrino J.-L., Sailly F., Mercier C., Moreel V. (2001). Étude des tracas quotidiens des étudiants: Liens avec la santé perçue. Ann. Médico-Psychol. Rev. Psychiatr..

[6] Lazarus R.S. (1984). Puzzles in the study of daily hassles. J. Behav. Med..

[7] Pritchard M.E., Wilson G.S., Yamnitz B. (2007). What Predicts Adjustment Among College Students? A Longitudinal Panel Study. J. Am. Coll. Health.

[8] Brougham R.R., Zail C.M., Mendoza C.M., Miller J.R. (2009). Stress, Sex Differences, and Coping Strategies among College Students. Curr. Psychol..

[9] DeLongis A., Folkman S., Lazarus R.S. (1988). The impact of daily stress on health and mood: Psychological and social resources as mediators. J. Personal. Soc. Psychol..

[10] Larsson G., Berglund A.K., Ohlsson A. (2016). Daily hassles, their antecedents and outcomes among professional first responders: A systematic literature review. Scand. J. Psychol..

[11] Shankland R., Kotsou I., Vallet F., Bouteyre E., Dantzer C., Leys C. (2019). Burnout in university students: The mediating role of sense of coherence on the relationship between daily hassles and burnout. High. Educ..

[12] Lavee Y., Ben-Ari A. (2008). The association of daily hassles and uplifts with family and life satisfaction: Does cultural orientation make a difference?. Am. J. Community Psychol..

[13] Diener E. (1984). Subjective well-being. Psychol. Bull..

[14] Diener E.D., Emmons R.A., Larsen R.J., Griffin S. (1985). The satisfaction with life scale. J. Personal. Assess..

[15] McCullough M.E., Emmons R.A., Tsang J.-A. (2002). The grateful disposition: A conceptual and empirical topography. J. Personal. Soc. Psychol..

[16] Emmons R.A., McCullough M.E. (2003). Counting blessings versus burdens: An experimental investigation of gratitude and subjective well-being in daily life. J. Personal. Soc. Psychol..

[17] Cregg D.R., Cheavens J.S. (2020). Gratitude interventions: Effective self-help? A meta-analysis of the impact on symptoms of depression and anxiety. J. Happiness Stud..

[18] Petrocchi N., Couyoumdjian A. (2016). The impact of gratitude on depression and anxiety: The mediating role of criticizing, attacking, and reassuring the self. Self Identity.

[19] Jans-Beken L., Jacobs N., Janssens M., Peeters S., Reijnders J., Lechner L., Lataster J. (2019). Gratitude and health: An updated review. J. Posit. Psychol..

[20] Dickens L.R. (2017). Using gratitude to promote positive change: A series of meta-analyses investigating the effectiveness of gratitude interventions. Basic Appl. Soc. Psychol..

[21] Cunha L.F., Pellanda L.C., Reppold C.T. (2019). Positive psychology and gratitude interventions: A randomized clinical trial. Front. Psychol..

[22] Wood A.M., Joseph S., Linley P.A. (2007). Coping style as a psychological resource of grateful people. J. Soc. Clin. Psychol..

[23] Lambert N.M., Fincham F.D., Stillman T.F. (2012). Gratitude and depressive symptoms: The role of positive reframing and positive emotion. Cogn. Emot..

[24] Vallerand R.J. (1989). Vers une méthodologie de validation trans-culturelle de questionnaires psychologiques: Implications pour la recherche en langue française. Can. Psychol. Psychol. Can..

[25] Caputo A. (2016). Italian translation and validation of the GQ-6. Int. J. Wellbeing.

[26] Sumi K. (2017). Reliability and construct validity of the Gratitude Questionnaire 6 Item Form (GQ 6) in a sample of Japanese college students. J. Posit. Sch. Psychol..

[27] Hudecek M.F.C., Blabst N., Morgan B., Lermer E. (2020). Measuring gratitude in germany: Validation study of the German version of the Gratitude Questionnaire-Six item form (GQ-6-G) and the Multi-Component Gratitude Measure (MCGM-G). Front. Psychol..

[28] Scheier M.F., Carver C.S., Bridges M.W. (1994). Distinguishing optimism from neuroticism (and trait anxiety, self-mastery, and self-esteem): A reevaluation of the life orientation test. J. Personal. Soc. Psychol..

[29] Trottier C., Mageau G., Trudel P., Halliwell W.R. (2008). Validation de la version canadienne-française du Life Orientation Test-Revised. Can. J. Behav. Sci..

[30] Vitaliano P.P., Russo J., Carr J.E., Maiuro R.D., Becker J. (1985). The Ways of Coping Checklist: Revision and psychometric properties. Multivar. Behav. Res..

[31] Cousson F., Bruchon-Schweitzer M., Quintard B., Nuissier J. (1996). Analyse multidimensionnelle d’une échelle de coping: Validation française de la W.C.C. (Ways of Coping Checklist). Psychol. Fr..

[32] Blais M.R., Vallerand R.J., Pelletier L.G., Brière N.M. (1989). L’échelle de satisfaction de vie: Validation canadienne-française du “Satisfaction with Life Scale”. Can. J. Behav. Sci..

[33] Gauthier J., Bouchard S. (1993). Adaptation canadienne-française de la forme révisée du State–Trait Anxiety Inventory de Spielberger. Can. J. Behav. Sci..

[34] Spielberger C.D. (1983). Manual for the State-Trait Anxiety Inventory (STAI).

[35] Fuhrer R., Rouillon F. (1989). La version française de l’échelle CES-D (Center for Epidemiologic Studies-Depression Scale). Description et traduction de l’échelle d’autoévaluation. Psychiatry Psychobiol..

[36] Radloff L.S. (1977). The CES-D Scale: A self-report depression scale for research in the general population. Appl. Psychol. Meas..

[37] Holm J.E., Holroyd K.A. (1992). The daily hassles scale (revised): Does it measure stress or symptoms?. Behav. Assess..

[38] Bono G., Mangan S., Fauteux M., Sender J. (2020). A new approach to gratitude interventions in high schools that supports student wellbeing. J. Posit. Psychol..

[39] Morgan B., Gulliford L., Kristjánsson K. (2014). Gratitude in the UK: A new prototype analysis and a cross-cultural comparison. J. Posit. Psychol..

[40] Morgan B., Gulliford L., Kristjánsson K. (2017). A new approach to measuring moral virtues: The Multi-Component Gratitude Measure. Personal. Individ. Differ..

[41] Navarro J.L., Tudge J.R.H. (2020). What is gratitude? Ingratitude provides the answer. Hum. Dev..

[42] Gulliford L., Morgan B., Kristjánsson K. (2013). Recent work on the concept of gratitude in philosophy and psychology. J. Value Inq..

